# Cardiovascular disease risks in younger versus older adult B‐cell non‐Hodgkin’s lymphoma survivors

**DOI:** 10.1002/cam4.3934

**Published:** 2021-05-12

**Authors:** Krista Ocier, Sarah Abdelaziz, Seungmin Kim, Kerry Rowe, John Snyder, Vikrant Deshmukh, Michael Newman, Alison Fraser, Ken Smith, Christina A. Porucznik, Kimberley Shoaf, Joseph B. Stanford, Catherine J. Lee, Mia Hashibe

**Affiliations:** ^1^ Division of Public Health Department of Family & Preventive Medicine University of Utah School of Medicine Salt Lake City UT USA; ^2^ Huntsman Cancer Institute Salt Lake City UT USA; ^3^ Intermountain Healthcare Salt Lake City UT USA; ^4^ University of Utah Health Sciences Center Salt Lake City UT USA; ^5^ Pedigree and Population Resource Population Sciences Huntsman Cancer Institute Salt Lake City UT USA; ^6^ Division of Hematology and Hematologic Malignancies Department of Internal Medicine University of Utah School of Medicine Salt Lake City UT USA; ^7^ Utah Cancer Registry University of Utah Salt Lake City UT USA

**Keywords:** accelerated aging, cardiovascular disease, long‐term health, non‐Hodgkin's lymphoma, survivorship

## Abstract

**Introduction:**

Young cancer survivors may be at increased risk of early‐onset chronic health conditions. The aim of this population‐based study is to estimate cardiovascular disease (CVD) risk among younger versus older B‐cell non‐Hodgkin's lymphoma (B‐NHL) survivors compared with their respective general population cohorts.

**Methods:**

B‐NHL survivors diagnosed from 1997 to 2015 in the Utah Cancer Registry were matched with up to five cancer‐free individuals on birth year, sex, and birth state, using the statewide Utah Population Database. Electronic medical records and statewide health care facility data were used to identify disease outcomes ≥5 years after cancer diagnosis. Cox Proportional Hazards models were used to estimate hazard ratios for B‐NHL survivors diagnosed at <65 years and ≥65 years old.

**Results:**

Younger B‐NHL survivors had higher relative risks than older cancer survivors of chronic rheumatic disease of the heart valves (HR = 4.14, 99% CI = 2.17–7.89; *P* value_heterogeneity_ = 0.004); peri‐, endo‐, and myocarditis (HR = 2.43, 99% CI = 1.38–4.28; *P* value_heterogeneity_ = 0.016); diseases of the arteries (HR = 1.63, 99% CI = 1.21–2.21; *P* value_heterogeneity_ = 0.044); and hypotension (HR = 2.44, 99% CI = 1.58–3.75; *P* value_heterogeneity_ = 0.048). B‐NHL survivors of both age groups had elevated relative risks of heart disease overall and congestive heart failure.

**Conclusion:**

Younger B‐NHL survivors had higher risks than older B‐NHL survivors of specific cardiovascular diseases compared to their respective general population cohorts.

## Graphical abstract

This population‐based study demonstrates elevated cardiovascular disease risks in B‐NHL survivors (<65 vs. ≥65 years old) relative to their general population cohorts, suggesting that early aging among younger B‐NHL survivors may warrant an assessment of long‐term health effects as part of cancer survivorship care.

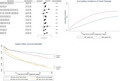

## 1 INTRODUCTION

Non‐Hodgkin's lymphoma (NHL) is the seventh‐most common cancer diagnosed in 2020^1^ and common hematological malignancy among adults aged 65 years and older,^2^ with B‐cell lymphoma accounting for 80%–90% of NHL cases.^3^ The 5‐year relative survival improved from 57.0% in the 1990 s to 67.5% in recent years,^4^, ^5^ with over 700,000 NHL survivors estimated in the USA in 2019.^6^ However, emerging evidence suggests that cancer survivors develop increased risks of early‐onset chronic health conditions compared to the general population,^7^, ^8^ including cardiovascular disease (CVD) outcomes, such as ischemic heart disease, stroke, and heart failure.^9^ This may be due to the cancer treatment that they underwent,^10^ further demonstrated by findings from two population‐based studies.^11^, ^12^

Prior studies on CVD risk in B‐NHL patients and survivors largely involved childhood/young adult cancer survivor studies^13^, ^14^, ^15^, ^16^ and clinical trials.^17^, ^18^, ^19^, ^20^, ^21^, ^22^, ^23^ The purpose of this study is to estimate the risk of developing CVD in younger adult versus older adult B‐NHL survivors compared to their respective general population cohorts in Utah. By comparing younger and older adult B‐NHL survivors to the general population, this may not only inform CVD risks related to B‐NHL but may also help identify if cancer treatment contributes to premature aging among younger B‐NHL survivors.

## 2 METHODS

### 2.1 Data collection

Eligibility criteria included individuals who were diagnosed with NHL (SEER ICD‐O‐3 codes: 33041–33042) from 1997 to 2015 in the Utah Cancer Registry (UCR). The NHL cohort was further stratified by age, with those diagnosed at <65 years old classified as the younger B‐NHL cohort and those diagnosed at ≥65 years old classified as the older B‐NHL cohort. A general population cohort of up to five cancer‐free individuals were matched to each NHL patient at the time of NHL diagnosis by sex, birth year, and birth state (Utah/not Utah) using the Utah Population Database (UPDB). The last follow‐up date was determined by UPDB through last contact with a number of statewide data sources, including driver license division, Utah birth certificate, and the Utah Department of Health (UDOH). Death dates were also captured nationwide using the Social Security Death Index and UCR records.

From an initial cohort of 5,326 NHL patients, exclusion criteria included: missing stage at diagnosis (n = 134), non B‐cell histology subtypes (n = 1,124), <5 years follow‐up to examine long‐term CVD risk (n = 1,918), and no match from the general population (n = 17), resulting in 2,129 B‐cell non‐Hodgkin's lymphoma (B‐NHL) survivors and 8,969 individuals from the general population. Studies using the UPDB data have been approved by the University of Utah's Resource for Genetic and Epidemiologic Research (oversight committee) and the University of Utah Institutional Review Board.

All participants were linked to the available healthcare data in the UPDB. Outcome data used for this study included statewide ambulatory surgery and inpatient data from the UDOH and electronic medical record data from Intermountain Healthcare and the University of Utah, the two largest health care organizations in the state. Utah is considered to have a minimal percentage of residents seeking healthcare out of the state^24^ and low out‐migration rate (2.9%).^25^

Outcome data included all available ICD‐9 diagnosis codes and diagnosis dates for diseases of the circulatory system. These include hypertension, diseases of the heart, cerebrovascular disease, diseases of the arteries, and diseases of the veins/lymphatics. The Clinical Classification Software (CCS) developed by the Health Cost and Utilization Project (HCUP) was used to categorize ICD‐9 codes into four levels of specificity (levels 1–4; Table S1). CVD outcomes diagnosed prior to the start of the analysis time period were considered prevalent cases and were thereby excluded.

Follow‐up time for incident cases of each CVD outcome was calculated from the initial lymphoma diagnosis of the B‐NHL survivor to the date of CVD diagnosis, last date of follow‐up, or date of death. Individuals without any CVD outcome were censored at the last follow‐up if that date fell within the analysis time period.

### 2.2 Statistical analysis

Chi‐squared tests were used to compare baseline characteristics between B‐NHL survivors and general population. Cox proportional hazards models were used to calculate hazard ratios for CVD outcomes between B‐NHL survivors diagnosed at <65 and ≥65 years old compared with their respective general population cohorts ≥5 years after cancer diagnosis. We used 99% confidence intervals to account for multiple testing due to the large number of CVD outcomes. Hazard ratios for CVD outcomes stratified by age at diagnosis were estimated, accounting for matching by sex, birth year, and birth state with the STRATA statement in SAS. We also adjusted for race/ethnicity, baseline body mass index (BMI), baseline Charlson Comorbidity Index (CCI), and smoking. We used the test for heterogeneity to compare hazard ratios for each CVD outcome in younger versus older B‐NHL survivors.

Cox proportional hazards models were also used to investigate CVD risk factors among B‐NHL survivors such as cancer treatment type, baseline smoking, baseline CCI, baseline BMI, and family history of heart disease. Types of cancer treatment consisted of chemotherapy, radiation, combination of chemotherapy and radiation, and hematopoietic cell transplantation (HCT), which includes both autologous and allogeneic transplants. We selected potential confounders to adjust on based on an assessment of the three properties of a confounder.^26^ The proportional hazards assumption was checked for each model using a test for nonzero slope of the Schoenfeld residuals versus time. Models that were in violation of the proportional hazards assumption were then tested with flexible parametric survival models with restricted cubic splines.

Baseline BMI values at least 1 year prior to B‐NHL diagnosis were calculated from driver's license records. For individuals with missing BMI, values were imputed using a linear regression model that included cancer diagnosis, baseline CCI, and race as covariates. We compared Cox regression models including those with and without imputed BMI to assure that inferences did not change due to the imputed BMI. We identified tobacco smokers with the ICD‐9 code for “tobacco use disorders” 305.1, ICD‐10 codes for nicotine dependence, and with CPT codes for tobacco cessation counseling based on the American Academy of Family Physicians coding guidelines.^27^

All statistical tests were two‐sided, and a *P* value of less than.05 was considered statistically significant to compare characteristics between cancer survivors and the general population and for the risk factor analyses among B‐NHL survivors. To estimate risk of CVD outcomes, we considered a *P* value of less than.01 as statistically significant.

## 3 RESULTS

Among 2,129 B‐NHL survivors matched to 8,969 cancer‐free individuals from the general population ≥5 years after cancer diagnosis, 54.6% of B‐NHL survivors were men (Table 1). Study participants were 7.5% Hispanic among the B‐NHL survivors and 6.1% Hispanic among the general population. At baseline, 60.4% of B‐NHL survivors had no preexisting comorbidity based on the CCI score, while 11.3% of B‐NHL survivors were smokers. Approximately 62.1% of B‐NHL survivors had a family history of heart disease. Although the individuals from the general population were matched on birth year, there appears to be a difference in the age distribution. The difference in age may have been caused by exclusion of individuals from the general population who had cancer after the matching took place.

**TABLE 1:** Demographic characteristics of B‐NHL survivors and general population cohort

	B‐NHL survivors	General population	Chi‐square *p* value
	n (%)	n (%)	
Sex			
Male	1,162 (54.6)	4,917 (54.8)	0.8398
Female	967 (45.4)	4,052 (45.2)	
Age^b^			
18–44 years old	328 (15.4)	1,492 (16.6)	0.0003
45–64 years old	951 (44.7)	4,267 (47.6)	
65–80 years old	748 (35.1)	2,912 (32.5)	
80+ years old	102 (4.8)	298 (3.3)	
Race			
White	2,079 (97.7)	8,439 (94.1)	<0.0001
Other	‐^a^(0.1)	210 (2.3)	
Unknown	48 (2.3)	320 (3.6)	
Ethnicity			
Non‐Hispanic	1,969 (92.5)	8,418 (93.9)	0.0201
Hispanic	160 (7.5)	551 (6.1)	
Vital status			
Alive	1,593 (74.8)	7,914 (88.2)	<0.0001
Dead	536 (25.2)	1,055 (11.8)	
Charlson comorbidity Index (CCI) at baseline			
0	1,286 (60.4)	6,266 (69.9)	<0.0001
1	444 (20.9)	1,619 (18.1)	
2+	399 (18.7)	1,084 (12.1)	
Body mass index (BMI) at baseline			
<18.5 kg/m^2^	22 (1.0)	93 (1.0)	0.0307
18–24.9 kg/m^2^	740 (34.8)	3,403 (37.9)	
25–29.9 kg/m^2^	911 (42.8)	3,737 (41.7)	
30+ kg/m^2^	456 (21.4)	1,736 (19.4)	
Smoking			
No	1,888 (88.7)	8,231 (91.8)	<0.0001
Yes	241 (11.3)	738 (8.2)	
Family history of heart disease^b^, ^c^			
No	806 (37.9)	3,160 (35.2)	0.0230
Yes	1,323 (62.1)	5,809 (64.8)	

^a^ Counts ≤11 are not shown per Utah Department of Health data suppression guidelines.

^b^ The age for the general population is the age at which the individual from the general population was matched to the B‐NHL patient. For lymphoma patients, the age was the age at cancer diagnosis.

^c^ Family history includes first‐, second‐, and third‐degree relatives.

Chemotherapy alone was the predominant mode of treatment among younger (41.5%) and older (38.7%) B‐NHL survivors, while surgery was not considered a curative treatment regimen for this cohort (Table 2).^28^ Approximately, 11.4% of younger B‐NHL survivors received HCT compared with 2.0% of older B‐NHL survivors. Of the histological subtypes, 43.5% of younger B‐NHL survivors and 44.2% of older B‐NHL survivors were diagnosed with diffuse large B‐cell lymphoma.

**TABLE 2:** Clinical characteristics of B‐NHL survivors who survived ≥5 years after cancer diagnosis

	<65 years old	≥65 years old
	n (%)	n (%)
Diagnosis year		
1997–2000	229 (17.9)	155 (18.2)
2001–2005	411 (32.1)	288 (33.9)
2006–2010	527 (41.2)	328 (38.6)
2011–2015	112 (8.8)	79 (9.3)
Cancer stage at diagnosis		
Localized	467 (36.5)	345 (40.6)
Regional	240 (18.8)	144 (16.9)
Distant	572 (44.7)	361 (42.5)
First Course Treatment		
No treatment	324 (25.3)	293 (34.5)
Chemotherapy	531 (41.5)	329 (38.7)
Radiation therapy	108 (8.4)	64 (7.5)
Chemotherapy + Radiation therapy	261 (20.4)	128 (15.1)
Unknown	55 (4.3)	36 (4.2)
Hematopoietic cell transplantation		
No	1,133 (88.6)	833 (98.0)
Yes	146 (11.4)	17 (2.0)
Aggressive B‐NHL subtypes		
Diffuse large B‐cell, NOS	498 (43.5)	344 (44.2)
Diffuse large B‐cell, immunoblastic, NOS	18 (1.6)	14 (1.8)
Diffuse mixed lymphoma	‐^a^	‐^a^(0.5)
Mediastinal large B‐cell lymphoma	‐^a^(0.9)	0
Burkitt lymphoma, NOS	32 (2.8)	‐^a^(0.3)
Follicular lymphoma, grade 3	73 (6.4)	41 (5.3)
Indolent B‐NHL subtypes		
Small B lymphocytic, NOS	60 (5.2)	69 (8.9)
Lymphoplasmacytic lymphoma	‐^a^(0.9)	‐^a^(1.3)
Mantle cell lymphoma	43 (3.8)	35 (4.5)
Splenic marginal zone B‐cell lymphoma	13 (1.1)	‐^a^
Marginal zone B‐cell lymphoma, NOS	134 (11.7)	112 (14.4)
Chronic lymphocytic leukemia/SLL	16 (1.4)	14 (1.8)
Follicular lymphoma, grade 2	111 (9.7)	60 (7.7)
Follicular lymphoma, grade 1	116 (10.1)	65 (8.3)
Cancer site		
Nodal	870 (68.0)	556 (65.4)
Extra nodal	409 (32.0)	294 (34.6)

^a^ Counts ≤11 are not shown per Utah Department of Health data suppression guidelines.

**FIGURE 1:**
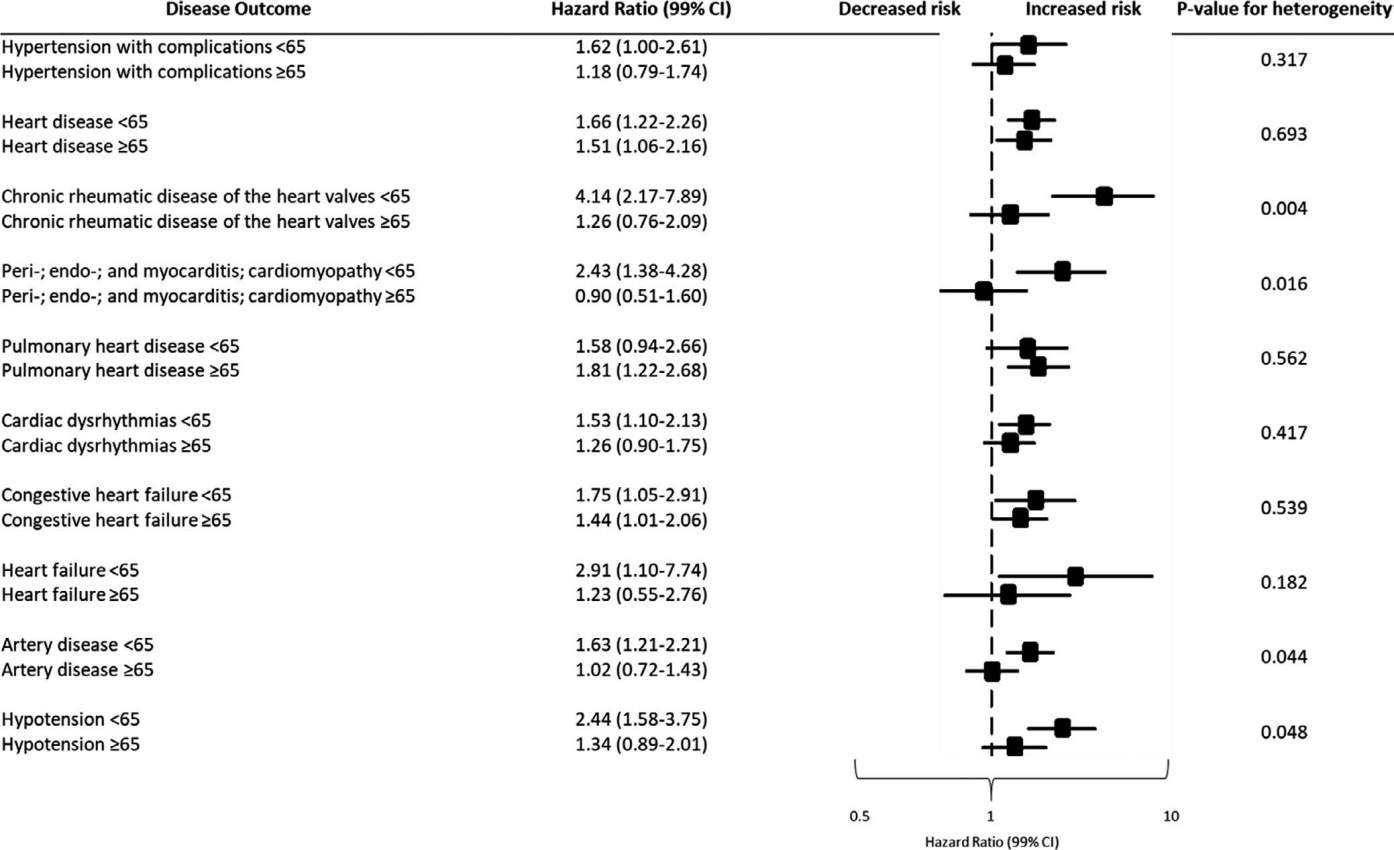
Cardiovascular disease risks between younger and older B‐NHL survivors ≥5 years after cancer diagnosis

Among all CVD outcomes that were investigated ≥5 years after cancer diagnosis (Figure 1), chronic rheumatic disease of the heart valves; peri‐, endo‐, and myocarditis; diseases of the arteries; and hypotension conferred higher relative risks in younger B‐NHL survivors than with older B‐NHL survivors relative to their respective general population cohorts ≥5 years after cancer diagnosis. No association was suggested among older B‐NHL survivors for these disease outcomes.

B‐NHL survivors had an elevated risk of diseases of the heart overall compared to the general population ≥5 years after cancer diagnosis (Table 3). Specifically, B‐NHL survivors had increased risks of congestive heart failure compared to their respective general population cohorts.

**TABLE 3:** Hazard ratios of diseases of the heart in B‐NHL survivors ≥5 years after cancer diagnosis compared to matched general population cohort, stratified by age

	<65 years old			≥65 years old			*p‐value* *
	General population	B‐NHL survivors		General population	B‐NHL survivors		
	n (%)	n (%)	HR (99%CI)	n (%)	n (%)	HR (99%CI)	
Diseases of the heart	606 (15.1)	145 (23.3)	1.66 (1.22–2.26)	438 (31.9)	98 (40.3)	1.51 (1.06–2.16)	0.693
Heart valve disorders	219 (4.0)	72 (6.5)	1.59 (1.04–2.42)	309 (11.4)	79 (12.0)	1.16 (0.79–1.71)	0.280
Chronic rheumatic disease of the heart valves	67 (1.2)	44 (3.6)	4.14 (2.17–7.89)	173 (5.7)	44 (5.7)	1.26 (0.76–2.09)	0.004
Peri‐, endo‐, and myocarditis; cardiomyopathy	101 (1.8)	46 (3.9)	2.43 (1.38–4.28)	156 (5.1)	31 (4.1)	0.90 (0.51–1.60)	0.016
Cardiomyopathy	75 (1.3)	35 (2.8)	2.32 (1.20–4.50)	115 (3.7)	27 (3.4)	1.01 (0.53–1.91)	0.077
Nonspecific chest pain	372 (8.0)	115 (12.9)	1.54 (1.10–2.18)	281 (12.5)	83 (15.0)	1.27 (0.87–1.87)	0.462
Pulmonary heart disease	152 (2.7)	50 (4.2)	1.58 (0.94–2.66)	234 (8.0)	83 (11.5)	1.81 (1.22–2.68)	0.683
Conduction disorders	117 (2.1)	41 (3.3)	1.84 (1.04–3.26)	226 (7.9)	68 (9.1)	1.30 (0.85–1.99)	0.339
Cardiac dysrhythmias	407 (8.0)	119 (12.0)	1.53 (1.10–2.13)	443 (20.2)	108 (21.4)	1.26 (0.90–1.75)	0.417
Atrial fibrillation	157 (2.8)	60 (4.9)	2.08 (1.30–3.33)	316 (11.6)	96 (14.2)	1.39 (0.97–2.01)	0.184
Congestive heart failure; nonhypertensive	171 (3.1)	58 (4.8)	1.74 (1.08–2.81)	332 (12.1)	105 (15.6)	1.51 (1.08–2.11)	0.634
Congestive heart failure	150 (2.7)	52 (4.3)	1.75 (1.05–2.91)	301 (10.9)	92 (13.6)	1.44 (1.01–2.06)	0.539
Heart failure	35 (0.6)	15 (1.2)	2.91 (1.10–7.74)	70 (2.2)	19 (2.3)	1.23 (0.55–2.76)	0.182

^a^ Models used the STRATA statement to account for matching factors and adjusted for race/ethnicity, baseline BMI, baseline CCI (excluding CVD‐related outcomes), and smoking.

^b^ The following outcomes were evaluated, but no elevated risk was observed: Nonrheumatic mitral valve disorders, nonrheumatic aortic valve disorders, other heart valve disorders, other peri‐, endo‐, and myocarditis, acute myocardial infarction, coronary atherosclerosis and other heart disease, angina pectoris, unstable angina, other acute and subacute forms of ischemic heart disease, coronary atherosclerosis, other forms of chronic heart disease, other and ill‐defined heart disease, atrioventricular block, bundle branch block, anomalous atrioventricular excitation, other conduction disorders, paroxysmal supraventricular tachycardia, paroxysmal ventricular tachycardia, atrial flutter, premature beats, sinoatrial node dysfunction, other cardiac dysrhythmias, cardiac arrest, and ventricular fibrillation.

* *P* value for statistical heterogeneity was calculated by using the test for heterogeneity to assess the difference in hazard ratios between younger and older cohort.

Younger B‐NHL survivors had increased relative risks of heart valve disorders, cardiomyopathy, nonspecific chest pain, conduction disorders, cardiac dysrhythmias, atrial fibrillation, and heart failure compared to the younger general population. Conversely, older B‐NHL survivors had an increased relative risk of pulmonary heart disease compared to the older general population cohort. In terms of specific relative risks between younger and older B‐NHL survivors, the younger cohort had higher relative risks of chronic rheumatic disease of the heart valves at 4.14‐fold (*P* value_heterogeneity_ = 0.004) and of peri‐, endo‐, and myocarditis at 2.43‐fold (*P* value_heterogeneity_ = 0.016) than older B‐NHL survivors.

Younger B‐NHL survivors had elevated risks of diseases of the veins/lymphatics, phlebitis and thrombophlebitis, and hemorrhoids compared to the general population ≥5 years after cancer diagnosis (Table 4). In terms of specific relative risks between younger and older B‐NHL survivors, the younger cohort had elevated risks of diseases of the arteries at 1.63‐fold (*P* value_heterogeneity_ = 0.044) and hypotension at 2.44‐fold (*P* value_heterogeneity_ = 0.048). Younger B‐NHL survivors also had an elevated risk of hypertension at borderline significance compared to the younger general population ≥5 years after cancer diagnosis (Table S2). However, this association was not observed in the older cohort. No significant associations were observed for cerebrovascular disease.

**TABLE 4:** Hazard ratios of diseases of the arteries and veins and lymphatics in B‐NHL survivors ≥5 years after cancer diagnosis compared to matched general population cohort, stratified by age

	<65 years old			≥65 years old			*p‐value*
	General population	B‐NHL survivors		General population	B‐NHL survivors		
	n (%)	n (%)	HR (99%CI)	n (%)	n (%)	HR (99%CI)	
Diseases of arteries, arterioles, and capillaries	498 (10.0)	148 (15.9)	1.63 (1.21–2.21)	513 (24.0)	103 (23.3)	1.02 (0.72–1.43)	0.044
Other circulatory disease [117.]	453 (8.9)	134 (13.8)	1.58 (1.16–2.16)	470 (20.6)	106 (20.2)	1.11 (0.79–1.54)	0.129
Hypotension	163 (2.9)	76 (6.5)	2.44 (1.58–3.75)	231 (7.8)	75 (10.4)	1.34 (0.89–2.01)	0.048
Diseases of veins and lymphatics	513 (11.5)	137 (17.1)	1.64 (1.43–1.88)	289 (13.4)	72 (15.5)	1.38 (0.92–2.07)	0.429
Phlebitis, thrombophlebitis, and thromboembolism	142 (2.6)	46 (4.1)	2.17 (1.71–2.75)	143 (4.9)	43 (6.2)	1.44 (0.87–2.39)	0.150
Hemorrhoids	416 (8.8)	117 (12.2)	1.70 (1.23–2.33)	168 (6.9)	42 (6.9)	1.23 (0.71–2.14)	0.320

^a^ Models used the STRATA statement to account for matching factors and adjusted for race/ethnicity, baseline BMI, baseline CCI (excluding CVD‐related outcomes), and smoking.

^b^ The following outcomes were evaluated, but no elevated risk was observed: Peripheral and visceral atherosclerosis, atherosclerosis of arteries of extremities, peripheral vascular disease unspecified, other peripheral and visceral atherosclerosis, aortic, peripheral, and visceral artery aneurysms, abdominal aortic aneurysm; without rupture, other aneurysm, aortic and peripheral arterial embolism or thrombosis, arterial embolism and thrombosis of lower extremity artery, other arterial embolism and thrombosis, other and unspecified circulatory disease, phlebitis and thrombophlebitis, other venous embolism and thrombosis, and varicose veins of lower extremity.

* *P value* for statistical heterogeneity was calculated by using the test for heterogeneity to assess the difference in hazard ratios between younger and older cohort.

Statistically significant predictors of heart disease for older B‐NHL survivors included chemotherapy and HCT, while hypercholesterolemia and hypertension at baseline were significant predictors among younger B‐NHL survivors (Table 5). Baseline cardiovascular comorbidities were significant risk factors of diseases of the arteries among younger B‐NHL survivors (Table S3).

**TABLE 5:** Risk factors for diseases of the heart among B‐NHL survivors ≥5 years after cancer diagnosis, stratified by age

	Diseases of the heart	
	<65 years old	≥65 years old
	HR (95% CI)	HR (95% CI)
Treatment type^b^		
No treatment	1.00	1.00
Chemotherapy	1.15 (0.72–1.83)	3.68 (1.92–7.04)*
Radiation therapy	0.58 (0.27–1.25)	0.99 (0.34–2.88)
Chemotherapy +Radiation therapy	0.88 (0.50–1.53)	2.40 (1.10–5.26)*
Hematopoietic cell transplantation^b^		
No	1.00	1.00
Yes	2.94 (1.73–5.01)	5.58 (1.75–17.77)*
Charlson comorbidity Index (CCI) at baseline^c^		
0	1.00	1.00
1	1.31 (0.80–2.14)	1.68 (0.87–3.24)
2+	2.61 (1.04–6.53)	1.39 (0.50–3.84)
Body mass index (BMI) at baseline^d^		
<18.5 kg/m^2^	3.02 (1.16–7.84)	‐
18–24.9 kg/m^2^	1.00	1.00
25–29.9 kg/m^2^	0.99 (0.68–1.44)	1.41 (0.90–2.22)
30+ kg/m^2^	1.08 (0.68–1.70)	1.02 (0.57–1.84)
Smoking		
No	1.00	1.00
Yes	1.56 (0.79–3.07)	0.80 (0.25–2.54)
Family history of heart disease		
No	1.00	1.00
Yes	1.28 (0.91–1.80)	1.68 (1.04–2.69)
Baseline hypercholesterolemia^e^		
No	1.00	1.00
Yes	1.84 (1.24–2.71)*	1.08 (0.68–1.73)
Baseline hypertension^e^		
No	1.00	1.00
Yes	2.24 (1.54–3.25)*	1.29 (0.83–1.98)

^a^ All models were adjusted for sex and race/ethnicity.

^b^ Additionally adjusted for baseline CCI (excluding CVD‐related outcome), baseline BMI, smoking, cancer stage at diagnosis, histology, and diagnosis year.

^c^ Additionally adjusted for smoking, baseline BMI, and diagnosis year.

^d^ Additionally adjusted for smoking, family history of heart disease, diagnosis year, and baseline CCI (excluding CVD‐related outcome).

^e^ Additionally adjusted for baseline CCI (excluding CVD‐related outcome), baseline BMI, and smoking.

* *P* values are statistically significant when assessing differences between younger and older B‐NHL survivors.

**FIGURE 2:**
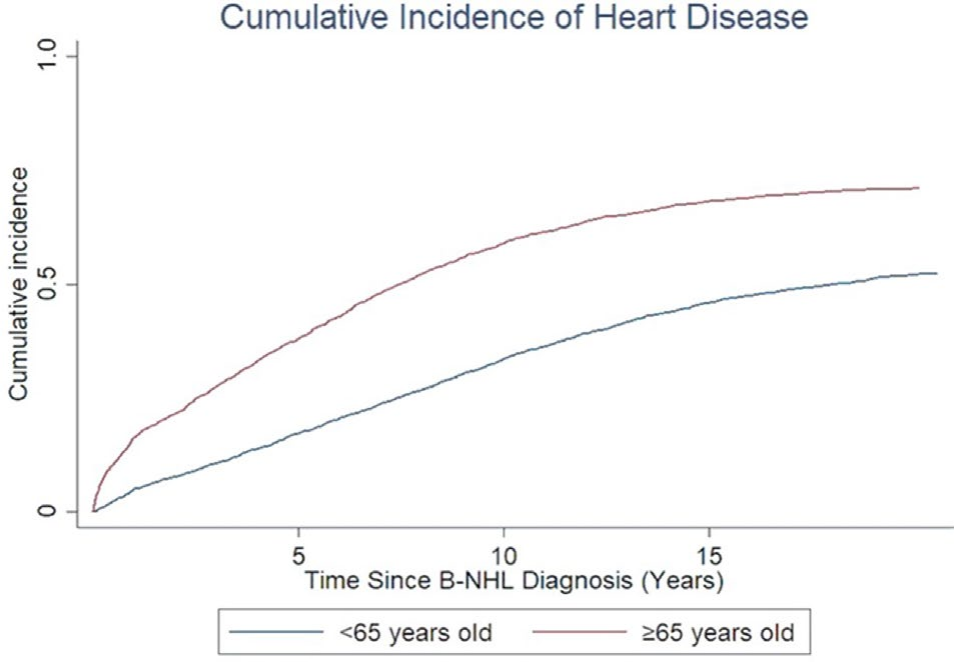
Cumulative incidence of heart disease among B‐NHL survivors

**FIGURE 3:**
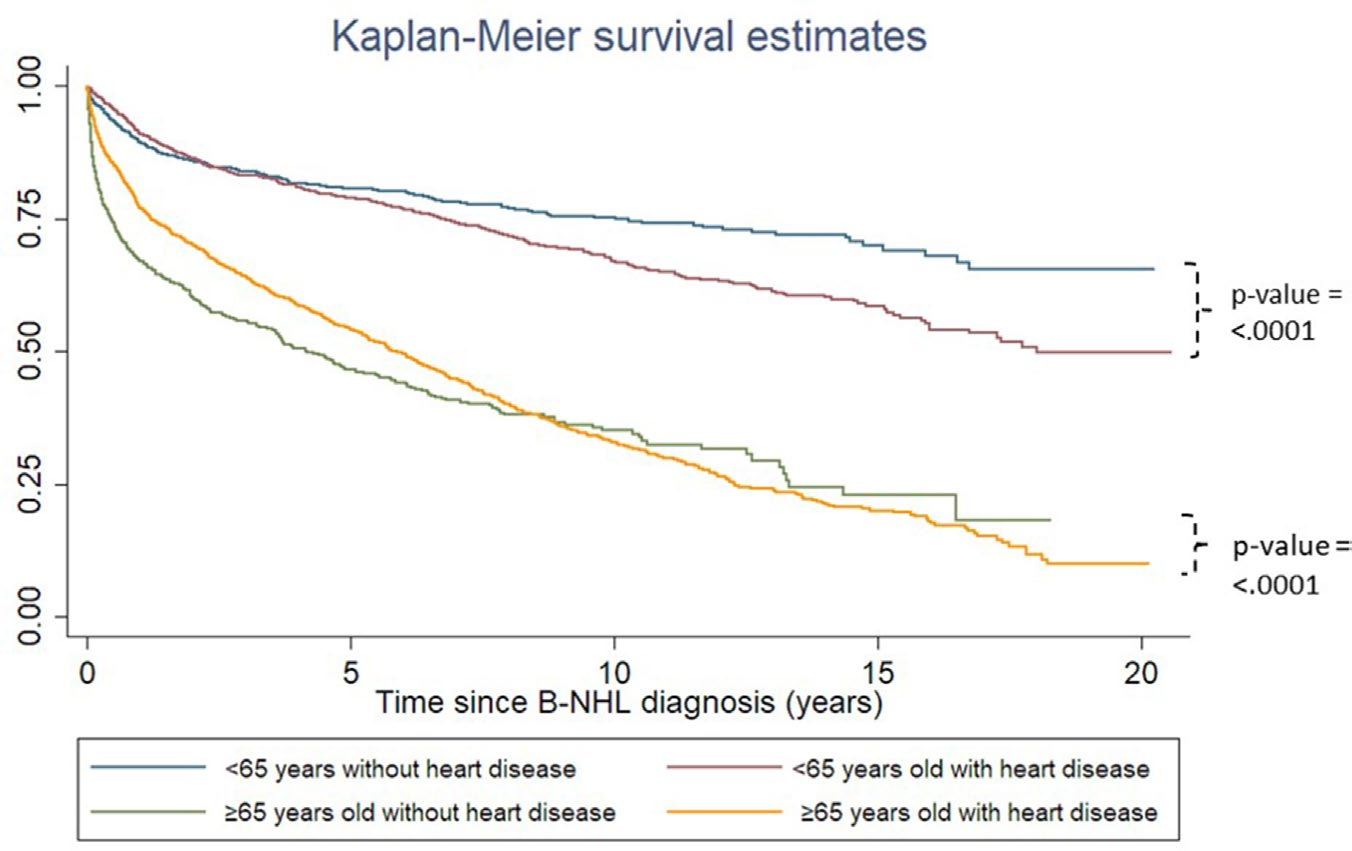
Survival curves of heart disease among B‐NHL survivors

The comparison of cumulative incidence curves for newly diagnosed diseases of the heart over time showed higher incidence among the older compared with the younger cohort (Figure 2). The comparison of Kaplan‐Meier survival curves further demonstrated that B‐NHL survivors with heart disease showed poorer survival outcomes compared with those without heart disease (Figure 3).

## 4 DISCUSSION

Our study is the first to examine risks of a range of CVD outcomes using ICD codes for B‐NHL survivors diagnosed at <65 vs. ≥65 years old compared with matched individuals from their respective general population cohorts in a large‐scale population‐based study. Younger B‐NHL survivors had higher relative risks of chronic rheumatic disease of the heart valves; peri‐, endo‐, and myocarditis; diseases of the arteries; and hypotension than older B‐NHL survivors. Risk factors of heart disease included baseline cardiovascular comorbidities among younger B‐NHL survivors and cancer treatment among older B‐NHL survivors.

While elevated risks of chronic rheumatic disease of the heart valves and peri‐, endo‐, and myocarditis were not observed in prior studies, this finding may help elucidate the biological implications related to B‐NHL, as B‐NHL is associated with a reduction in normal B cells fighting off an infection. Specifically, rheumatic heart disease develops when rheumatic fever is left untreated and is associated with group‐A streptococcal pharyngitis often found in children and young adults,^29^ whereas peri‐, endo‐, and myocarditis can evolve from a viral or bacterial infection that results in inflammation of the heart muscle.^30^

In terms of other CVD outcomes in our study, younger B‐NHL survivors developed risks of cardiomyopathy, cardiac dysrhythmias, and heart failure compared to the younger general population, suggesting an association between B‐NHL treatment and CVD‐related late effects. A self‐reported prospective study including 7.8% (n=75) of 957 adult NHL patients developed heart failure, myocardial infarction, arrhythmia, pericarditis, and valvular heart disease,^31^ and a community‐based retrospective cohort study including 1,524 NHL survivors observed a 1.41‐fold CVD risk overall, with a 1.35‐fold risk of ischemic heart disease.^9^ While a population‐based study in Netherlands of 2,184 NHL survivors observed no CVD risk compared to their cancer‐free cohort,^32^ a UK population‐based study comparing 4,423 NHL survivors to their respective general population cohort observed elevated risks of heart failure or cardiomyopathy in the younger than older cohorts: <60 years old (RR = 4.38, 95% CI = 2.74–7.01), 60–79 years old (RR = 1.96, 95% CI = 1.62–2.38), and ≥80 years old (RR = 1.40, 95% CI = 1.04–1.88).^12^ These prior findings are comparable with our study as we also observed elevated risks of heart and artery diseases in the B‐NHL age cohorts.

We observed a 2.45‐fold risk of hypotension in younger B‐NHL survivors compared to older B‐NHL survivors in our study. While hypotension is not a common outcome of B‐NHL in prior studies, it would be beneficial to understand if this is a common occurrence among B‐NHL patients, as some case findings^33^, ^34^ observed an elevated risk of hypotension during and after cancer treatment.

In terms of risk factors, our finding corroborates with prior studies as cancer treatment^31^, ^35^, ^36^, ^37^, ^38^, ^39^ and preexisting hypertension^36^, ^40^ were associated with elevated risks of heart failure. Despite increased relative risks of specific diseases of the heart among the younger cohort, cumulative incidence of heart disease was higher in the older cohort. This supports conclusions from prior studies in which absolute risks for CVD rose with increasing age due to their baseline risks.^12^ The survival curves further illustrate how treatment regimens may impact survival outcomes of B‐NHL patients with respect to heart disease. A possible explanation as to why the survival curves crossed between the older cohorts with and without heart disease may be that the ≥65‐year‐old group with heart disease were deteriorating more steadily over time, while the ≥65‐year‐old group without heart disease received aggressive treatment initially but then stabilized over time. This may suggest that older adults should not be precluded from receiving aggressive treatment regimens.

Strengths of this study include the large sample size, which provided sufficient power to examine a large number of outcomes. In addition, our follow‐up period of ≥5 years after cancer diagnosis focuses on the long‐term health implications of B‐NHL survivors and is less susceptible to surveillance bias since cancer patients will be using the health care more than the general population when first diagnosed and for years after. The data used in the study incorporate medical records from the state's two largest health care providers as well as statewide ambulatory surgery and inpatient data, which provide comprehensive medical record data for a large number of individuals. In contrast to cancer survivor studies that rely on self‐reports of the disease, our study is less susceptible to survival bias because we used long‐term health records as the source for disease diagnoses.

This study also has a number of limitations. While we utilized comprehensive electronic medical record data from the two largest statewide health care systems, there is the possibility that study participants could have been diagnosed with cardiovascular outcomes in hospitals and clinics not covered by data sources. However, our data sources also include statewide records from the UPDB and UDOH; thus, the majority of the population was covered. Another limitation of this study is that some subjects had missing baseline BMI data, which was addressed by imputation of BMI values. Smoking was identified using ICD codes, which may be a limitation because only heavy smokers or complications due to smoking were evaluated. However, our results did show that smoking is a risk factor for artery disease among B‐NHL survivors. Treatment data were limited to broad categories and did not include type of drug, dosage, specific chemotherapy cycles, and duration of treatment.

In conclusion, we observed elevated relative risks of specific long‐term CVD outcomes in younger B‐NHL survivors compared with their older counterparts. Potential reasons for the increased risks include treatment‐associated side effects and presence of preexisting comorbidities, suggesting a need for monitoring and management of these outcomes in young B‐NHL survivors. Although these increased disease risks suggest some early aging for younger B‐NHL survivors, most CVD relative risks were not different between the two age groups. Future studies specifically observing these CVD outcomes prospectively are warranted.

5

## Supporting information

Table S1‐S3

## Data Availability

The data that support the findings of this study can be accessed through approvals with the Resource for Genetic and Epidemiologic Research Committee (RGE), the oversight committee for the UPDB and IRB.
