# Supplementary material for: Cardiovascular disease risks in younger versus older adult B‐cell non‐Hodgkin’s lymphoma survivors
Source: Cancer Med. 2021 May 12;10(12):4117–26. doi: 10.1002/cam4.3934 (PMC8209610; doi:10.1002/cam4.3934)
Supplement: Supplementary file 1 — Table S1‐S3 [file CAM4-10-4117-s001.pdf]

Supplemental Table 1. List of ICD-9 codes categorized by the Health Cost and Utilization Project (HCUP) into multi-level groupings of cardiovascular outcomes

CCS Level	Cardiovascular outcome	ICD-9 codes
7	Diseases of the circulatory system	
7.1	Hypertension	
7.1.1	Essential hypertension [98.]	4011 4019
7.1.2	Hypertension with complications and secondary hypertension [99.]	
7.1.2.1	Hypertensive heart and/or renal disease	40200 40201 40210 40211 40290 40291 4030 40300 40301 4031 40310 40311 4039 40390 40391 4040 40400 40401 40402 40403 4041 40410 40411 40412 40413 4049 40490 40491 40492 40493
7.1.2.2	Other hypertensive complications	4010 40501 40509 40511 40519 40591 40599 4372
7.2	Diseases of the heart	
7.2.1	Heart valve disorders [96.]	
7.2.1.1	Chronic rheumatic disease of the heart valves	3940 3941 3942 3949 3950 3951 3952 3959 3960 3961 3962 3963 3968 3969 3970 3971 3979
7.2.1.2	Nonrheumatic mitral valve disorders	4240
7.2.1.3	Nonrheumatic aortic valve disorders	4241
7.2.1.4	Other heart valve disorders	4242 4243 42490 42491 42499 7852 7853 V422 V433
7.2.2	Peri-; endo-; and myocarditis; cardiomyopathy (except that caused by TB or STD)	
7.2.2.1	Cardiomyopathy	4250 4251 42511 42518 4252 4253 4254 4257 4258 4259
7.2.2.2	Other peri-; endo-; and myocarditis	03282 03640 03641 03642 03643 07420 07421 07422 07423 11281 11503 11504 11513 11514 11593 11594 1303 3910 3911 3912 3918 3919 3920 393 3980 39890 39899 4200 42090 42091 42099 4210 4211 4219 4220 42290 42291 42292 42293

		42299<br>4230 4231 4232 4233 4238 4239 4290
7.2.3	Acute myocardial infarction [100.]	4100 41000 41001 41002 4101 41010 41011 41012 4102 41020<br>41021 41022 4103 41030 41031 41032 4104 41040 41041 41042<br>4105 41050 41051 41052 4106 41060 41061 41062 4107 41070<br>41071 41072 4108 41080 41081 41082 4109 41090 41091 41092
7.2.4	Coronary atherosclerosis and other heart disease [101.]	41406
7.2.4.1	Angina pectoris	4130 4131 4139
7.2.4.2	Unstable angina (intermediate coronary syndrome)	4111
7.2.4.3	Other acute and subacute forms of ischemic heart disease	4110 4118 41181 41189
7.2.4.4	Coronary atherosclerosis	4140 41400 41401 4142 4143 4144 V4582
7.2.4.5	Other forms of chronic heart disease	412 4148 4149 V4581
7.2.5	Nonspecific chest pain [102.]	78650 78651 78659
7.2.6	Pulmonary heart disease [103.]	4150 4151 41512 41513 41519 4160 4161 4162 4168 4169 4170 4171 4178 4179 V1255
7.2.7	Other and ill-defined heart disease [104.]	41410 41411 41412 41419 4291 4292 4293 4295 4296 42971 42979 42981 42982 42983 42989 4299
7.2.8	Conduction disorders [105.]	
7.2.8.1	Atrioventricular block	4260 42610 42611 42612 42613
7.2.8.2	Bundle branch block	4262 4263 4264 42650 42651 42652 42653 42654
7.2.8.3	Anomalous atrioventricular excitation	4267
7.2.8.4	Other conduction disorders	4266 42681 42682 42689 4269 V450 V4500 V4501 V4502 V4509<br>V533 V5331 V5332 V5339
7.2.9	Cardiac dysrhythmias [106.]	

7.2.9.1	Paroxysmal supraventricular tachycardia	4270
7.2.9.2	Paroxysmal ventricular tachycardia	4271
7.2.9.3	Atrial fibrillation	42731
7.2.9.4	Atrial flutter	42732
7.2.9.5	Premature beats	42760 42761 42769
7.2.9.6	Sinoatrial node dysfunction	42781
7.2.9.7	Other cardiac dysrhythmias	4272 42789 4279 7850 7851
7.2.10	Cardiac arrest and ventricular fibrillation [107.]	42741 42742 4275
7.2.11	Congestive heart failure; nonhypertensive [108.]	42820 42821 42822 42823 42830 42831 42832 42833 42840 42841 42842 42843
7.2.11.1	Congestive heart failure	4280
7.2.11.2	Heart failure	39891 4281 4289
7.3	Cerebrovascular disease	
7.3.1	Acute cerebrovascular disease [109.]	
7.3.1.1	Intracranial hemorrhage	430 431 4320 4321 4329
7.3.1.2	Occlusion of cerebral arteries	43301 43311 43321 43331 43381 43391 4340 43400 43401 4341 43410 43411 4349 43490 43491
7.3.1.3	Acute but ill-defined cerebrovascular accident	34660 34661 34662 34663 436
7.3.2	Occlusion or stenosis of precerebral arteries [110.]	4330 43300 4331 43310 4332 43320 4333 43330 4338 43380 4339 43390
7.3.3	Other and ill-defined cerebrovascular disease [111.]	4370 4371 4373 4374 4375 4376 4377 4378 4379
7.3.4	Transient cerebral ischemia [112.]	4350 4351 4352 4353 4358 4359
7.3.5	Late effects of cerebrovascular disease [113.]	438 4380 43810 43811 43812 43813 43814 43819 43820 43821 43822 43830 43831 43832 43840 43841 43842 43850 43851 43852 43853 4386 4387 43881 43882 43883 43884 43885 43889 4389

7.4	Diseases of arteries; arterioles; and capillaries	
7.4.1	Peripheral and visceral atherosclerosis [114.]	
7.4.1.1	Atherosclerosis of arteries of extremities	4402 44020 44021 44022 44023 44029 4404
7.4.1.2	Peripheral vascular disease unspecified	4439
7.4.1.3	Other peripheral and visceral atherosclerosis	4400 4401 4408 4409 5570 5571 5579
7.4.2	Aortic; peripheral; and visceral artery aneurysms [115.]	44321 44322 44323 44324 44329
7.4.2.1	Abdominal aortic aneurysm; without rupture	4414 44772
7.4.2.2	Other aneurysm	4410 44100 44101 44102 44103 4411 4412 4413 4415 4416 4417 4419 4420 4421 4422 4423 44281 44282 44283 44284 44289 4429 44770 44771 44773
7.4.3	Aortic and peripheral arterial embolism or thrombosis [116.]	44501 44502 44581 44589
7.4.3.1	Arterial embolism and thrombosis of lower extremity artery	44422
7.4.3.2	Other arterial embolism and thrombosis	4440 44401 44409 4441 44421 44481 44489 4449
7.4.4	Other circulatory disease [117.]	
7.4.4.1	Hypotension	4580 4581 4588 4589
7.4.4.2	Other and unspecified circulatory disease	4430 4431 44381 44382 44389 4460 4461 4462 44620 44621 44629 4463 4464 4465 4466 4467 4470 4471 4472 4473 4474 4475 4476 4478 4479 4480 4481 4489 4590 45989 4599 7859 79430 79431 79439 7962 V125 V1250 V1253 V1254 V1259 V151 V421 V432 V4321 V4322 V434 V717
7.5	Diseases of veins and lymphatics	
7.5.1	Phlebitis; thrombophlebitis and thromboembolism [118.]	
7.5.1.1	Phlebitis and thrombophlebitis	4510 45111 45119 4512 45181 45182 45183 45184 45189 4519 V1252
7.5.1.2	Other venous embolism and thrombosis	452 4530 4531 4532 4533 45340 45341 45342 45350 45351 45352 4536 45371 45372 45373 45374 45375 45376 45377 45379 4538 45381 45382 45383 45384 45385 45386 45387

		45389 4539<br>V1251
7.5.2	Varicose veins of lower extremity [119.]	4540 4541 4542 4548 4549
7.5.3	Hemorrhoids [120.]	4550 4551 4552 4553 4554 4555 4556 4557 4558 4559
7.5.4	Other diseases of veins and lymphatics [121.]	4563 4564 4565 4566 4568 4570 4571 4572 4578 4579<br>4591 45910 45911 45912 45913 45919 4592 45930<br>45931 45932<br>45933 45939 45981

Supplemental Table 2. Hazard ratios of hypertension and cerebrovascular disease ≥5 years after cancer diagnosis in B-NHL survivors compared to matched general population cohort, stratified by age

	<65 years old			≥65 years old			
	**General population n (%)**	**B-NHL survivors n (%)**	**HR (99%CI)**	**General population n (%)**	**B-NHL survivors n (%)**	**HR (99%CI)**	***P-value***
Hypertension	690 (16.8)	138 (17.8)	1.06 (0.79-1.43)	379 (32.5)	68 (27.8)	0.83 (0.52-1.31)	0.383
Essential hypertension	686 (16.6)	138 (17.5)	1.05 (0.78-1.42)	365 (30.8)	66 (26.5)	0.84 (0.53-1.34)	0.693
Hypertension with complications and secondary hypertension	184 (3.3)	57 (4.8)	1.62 (1.00-2.61)	298 (10.5)	70 (9.7)	1.18 (0.79-1.74)	0.429
Cerebrovascular disease	205 (3.7)	55 (4.5)	1.10 (0.67-1.80)	309 (11.5)	77 (11.0)	1.09 (0.73-1.61)	0.977
Acute cerebrovascular disease	100 (1.8)	27 (2.2)	1.19 (0.60-2.36)	183 (6.2)	47 (6.1)	1.07 (0.64-1.78)	0.807
Transient cerebral ischemia	57 (1.0)	23 (1.8)	1.56 (0.69-3.52)	113 (3.8)	14 (1.8)	0.63 (0.28-1.42)	0.122

*P-value for statistical heterogeneity was calculated by using the test for heterogeneity to assess the difference in hazard ratios between younger and older cohort

†Models used the STRATA statement to account for matching factors and adjusted for race/ethnicity, baseline BMI, baseline CCI, and smoking

‡The following outcomes were evaluated, but no elevated risk was observed: Hypertensive heart and/or renal disease, other hypertensive complications, intracranial hemorrhage, occlusion of cerebral arteries, acute but ill-defined cerebrovascular accident, occlusion or stenosis of precerebral arteries, other and ill-defined cerebrovascular disease, late effects of cerebrovascular disease

Supplemental Table 3. Risk factors for diseases of the arteries among B-NHL survivors ≥5 years after cancer diagnosis, stratified by age

	Diseases of the arteries	
	<65 years old HR (95% CI)	≥65 years old HR (95% CI)
**Treatment type‡**		
No treatment	1.00	1.00
Chemotherapy	1.33 (0.85-2.08)	1.86 (1.03-3.36)
Radiation therapy	0.60 (0.28-1.28)	1.92 (0.72-5.13)
Chemotherapy + Radiation therapy	0.72 (0.40-1.29)	1.10 (0.52-2.35)
**Hematopoietic cell transplantation‡**		
No	1.00	1.00
Yes	2.12 (1.29-3.46)	2.76 (0.72-10.56)
**Charlson Comorbidity Index (CCI) at baseline§**		
0	1.00	1.00
1	1.65 (1.09-2.49)	1.04 (0.61-1.78)
2+	1.77 (0.85-3.70)	1.10 (0.40-3.07)
**Body mass index (BMI) at baseline¶**		
<18.5 kg/m^2	0.97 (0.23-4.08)	1.07 (0.14-7.95)
18-24.9 kg/m^2	1.00	1.00
25-29.9 kg/m^2	1.13 (0.78-1.64)	1.11 (0.70-1.77)
30+ kg/m^2	1.23 (0.79-1.93)	1.09 (0.63-1.87)
**Smoking**		
No	1.00	1.00
Yes	1.55 (0.88-2.75)	1.95 (1.00-3.79)
**Family history of heart disease**		
No	1.00	1.00
Yes	1.17 (0.83-1.63)	1.17 (0.76-1.79)
**Baseline hypercholesterolemia#**		
No	1.00	1.00
Yes	1.56 (1.10-2.22)*	1.01 (0.67-1.53)
**Baseline hypertension#**		
No	1.00	1.00
Yes	1.76 (1.25-2.49)*	1.32 (0.87-2.00)

†All models were adjusted for sex, race/ethnicity
‡Additionally adjusted for baseline CCI, baseline BMI, smoking, cancer stage at diagnosis, histology, diagnosis year
§Additionally adjusted for smoking, baseline BMI, diagnosis year
¶Additionally adjusted for smoking, family history of heart disease, diagnosis year, baseline CCI
#Additionally adjusted for baseline CCI, baseline BMI, and smoking
**P* values are statistically significant when assessing differences between younger and older B-NHL survivors
